# Differential mechanisms of continuous theta burst stimulation and low-frequency repetitive transcranial magnetic stimulation for chronic tinnitus: preliminary efficacy dissociation by local focalization vs. network dispersion

**DOI:** 10.3389/fneur.2026.1921227

**Published:** 2026-09-30

**Authors:** Mengxue Zhang, Li Qin, Enli Jin, Chaoqi Shi, Jiayu Ni, Jiaying Wang, Siqi Gao, Yiqi Ma, Xiao Li, Yuan Li

**Affiliations:** 1 Hangzhou Normal University, Hangzhou, China; 2 Deqing Hospital of Hangzhou Normal University, Huzhou, China; 3 Hangzhou Ninth People's Hospital, Hangzhou, China; 4 Zhejiang Cancer Hospital, Hangzhou, China; 5 Department of Otorhinolaryngology, Affiliated Hospital of Hangzhou Normal University, Hangzhou, China; 6 The Center for Caribbean Studies at Hangzhou Normal University, Hangzhou, China

**Keywords:** brain network, chronic subjective tinnitus, continuous theta burst stimulation, disease duration, functional magnetic resonance imaging, repetitive transcranial magnetic stimulation

## Abstract

**Objective:**

This study aimed to preliminarily compare clinical efficacy and neural mechanisms of continuous theta-burst stimulation (cTBS) and low-frequency repetitive transcranial magnetic stimulation (rTMS) targeting the auditory cortex in chronic subjective tinnitus. Differential effects on tinnitus loudness and distress, as well as the moderating role of disease duration, were explored.

**Methods:**

Twenty-nine patients were randomly assigned to cTBS (*n* = 16) or rTMS (*n* = 13) groups. Neuronavigation-guided stimulation was applied to the individualized auditory cortex peak activation site. Outcome measures included the Tinnitus Handicap Inventory (THI) and Visual Analog Scale (VAS) at baseline, post-treatment, 1-month, and 3-month follow-ups. Resting-state fMRI was used to analyze amplitude of low-frequency fluctuations (ALFF) and regional homogeneity (ReHo). Functional connectivity (FC) analysis was performed to explore the network-level changes.

**Results:**

Clinically, cTBS was associated with a rapid reduction in VAS-rated tinnitus loudness (*p* = 0.004), while low-frequency rTMS demonstrated sustained improvements in THI scores up to the 3-month follow-up (*p* = 0.007). Mixed-model ANCOVA yielded significant time-by-group interactions for THI and VAS, verifying different therapeutic profiles between cTBS and rTMS. Resting-state fMRI uncovered separable brain plasticity: cTBS induced focal changes in left auditory cortex, whereas rTMS remodeled extensive limbic-cognitive networks. Two-way ANCOVA confirmed shared left prefrontal interaction signals, supporting stronger prefrontal suppression by cTBS. Baseline tinnitus duration was negatively correlated with 3-month THI improvement rates. Collectively, clinical and dual resting-state imaging results identified separable therapeutic and neuromodulatory signatures of cTBS and low-frequency rTMS.

**Conclusion:**

Our exploratory results suggest that cTBS may facilitate a rapid reduction in tinnitus loudness possibly through focal auditory modulation, while low-frequency rTMS eases persistent tinnitus distress through large-scale network remodeling. Our findings provide preliminary evidence for the divergent prefrontal inhibitory profiles of the two inhibitory protocols. Shorter tinnitus duration predicts better therapeutic outcomes, highlighting the value of early intervention. These exploratory results provide evidence for personalized TMS-based neuromodulation in chronic tinnitus.

## 1 Introduction

Tinnitus is broadly categorized into subjective and objective subtypes. Objective tinnitus originates from physical internal sound-generating sources that can be detected by an examiner, whereas subjective tinnitus, the focus of the present work, refers to the perception of sound without any identifiable external or internal physical sound source (1). Subjective tinnitus represents the far more prevalent form, affecting approximately 10–15% of the general population, with increasing prevalence with age (2). Tinnitus lasting more than 6 months is defined as chronic. Although tinnitus is conventionally regarded primarily as an otorhinolaryngological symptom, accumulating evidence indicates that diverse systemic contributors also participate in its onset and persistence beyond inner-ear pathology (3). It is frequently comorbid with anxiety, depression, insomnia, and other psychological disorders (4, 5). These comorbidities severely reduce patients’ quality of life and pose major challenges for clinical intervention. Current mainstream treatments include sound therapy, cognitive behavioral therapy (CBT), tinnitus retraining therapy (TRT), and masking therapy, which mainly focus on compensation and habituation, with limited effects on core tinnitus symptoms in some patients (6).

Given these limitations, non-invasive neuromodulation has emerged as a promising intervention for chronic tinnitus (2). Transcranial Magnetic Stimulation (TMS) is one of a non-invasive neuromodulation techniques that regulates cortical neuronal excitability through induced electrical currents (7). Common protocols include low-frequency (1 Hz), high-frequency (10 Hz), and burst stimulation patterns (8). While 1 Hz low-frequency rTMS is established as a clinical guideline-recommended protocol for inhibiting auditory cortex hyperexcitability (9), its therapeutic application is constrained by notable practical and efficacy-related limitations. Standard clinical regimens require 20-min stimulation per session delivered over repeated visits across a two-week treatment course, imposing substantial time-related burden for patients. Furthermore, clinical outcomes are characterized by high inter-individual response variability and overall moderate therapeutic effect sizes in tinnitus cohorts (10). Continuous theta burst stimulation (cTBS) offers a compelling alternative due to its superior temporal efficiency; by mimicking endogenous theta rhythms, it induces robust long-term depression (LTD)-like effects through rapid GABAergic modulation (11). Although cTBS has been traditionally favored for probing immediate cortical excitability changes or neurotransmitter dynamics (12), its potential to provide sustained, clinically meaningful improvements in chronic tinnitus, comparable to those of standard rTMS, remains to be systematically investigated.

Given that these two protocols operate on different physiological time-scales—with cTBS potentially exerting a more rapid, focal inhibitory impact while rTMS facilitates broader, cumulative network remodeling—a head-to-head comparison is warranted. Such a study is vital for determining whether the high efficiency of cTBS in modulating neural synchrony translates into long-term relief for tinnitus-related distress, or if the two techniques serve distinct, complementary clinical roles.

Tinnitus duration may serve as a potential prognostic factor of treatment-related neuroplasticity and therapeutic outcomes among patients with chronic tinnitus. Previous studies suggest that prolonged tinnitus may lead to the spread of pathological neural activity from the auditory network to the whole-brain limbic-cognitive network, forming a more consolidated pathological state (13). All participants in this study had chronic tinnitus, and stimulation protocols were not tailored according to individual disease duration. Our primary aim was to compare therapeutic effects between cTBS and rTMS for tinnitus relief. As an additional exploratory post-hoc analysis, we further examined whether baseline tinnitus duration could predict therapeutic responses across both neuromodulation groups (14).

This preliminary study used rs-fMRI to compare the clinical efficacy and neural mechanisms of cTBS and low-frequency rTMS for chronic subjective tinnitus, and explored the correlation between disease duration and treatment response, aiming to provide exploratory evidence for personalized and precise tinnitus treatment.

## 2 Materials and methods

### 2.1 Participants

Sample size was estimated *a priori* using G*Power 3.1.9.7, based on the 2 (group) × 4 (time) mixed-design analysis of variance (ANOVA) (medium effect size *f* = 0.25, *α* = 0.05, power = 0.80). The required total sample size was 24, and 29 patients were enrolled considering a 20% dropout rate (15).

This study recruited 29 right-handed patients with chronic subjective tinnitus (duration ≥ 6 months, aged 18–65 years) from the Department of Otorhinolaryngology, Affiliated Hospital of Hangzhou Normal University (January 2022–December 2024). Patients were allocated to the rTMS group (*n* = 13) and cTBS group (*n* = 16) using simple randomization with a computer-generated random number list. Allocation sequences were generated offline and concealed from the research staff responsible for participant screening and enrolment. Unequal group sizes arose as a natural outcome of this randomization process among the 29 enrolled subjects (16). Inclusion criteria: normal hearing or mild-to-moderate sensorineural hearing loss (pure-tone average threshold ≤ 55 dB HL; mild: 26–40 dB HL, moderate: 41–55 dB HL); Exclusion criteria: pulsatile tinnitus, Ménière’s disease, otosclerosis, acoustic neuroma, severe hyperacusis, epilepsy, brain trauma, severe mental disorders, use of anxiolytics, antidepressants, or sedatives in the past 3 months, and MRI contraindications. This study was approved by the Ethics Committee of the Affiliated Hospital of Hangzhou Normal University (approval number: 2023(E2)-HS-068, approval date: May 18, 2023). All participants provided written informed consent prior to enrolment.

### 2.2 Scale assessment

All patients completed clinical assessments at four time points: baseline before treatment, on the day of treatment completion, and at 1-month and 3-month follow-ups. Assessment tools included the Tinnitus Handicap Inventory (THI) and the Visual Analogue Scale (VAS). The THI was used to assess the comprehensive impact of tinnitus on patients’ emotions, function, and catastrophic-thinking domains across three subscales: functional, emotional, and catastrophic. Total scores range from 0 and 100, with established severity stratification: 0–16 = slight, 18–36 = mild, 38–56 = moderate, 58–76 = severe, 78–100 = catastrophic; higher scores indicate greater tinnitus-related disability. The VAS was used to assess patients’ instantaneous subjective loudness of tinnitus at each time point, with scores ranging from 0 to 10. Post-treatment behavioural was acquired on the same day after completion of the full 10-session stimulation course. To analyze the relationship between disease duration and efficacy, the THI improvement rate at 3 months post-treatment was calculated using the formula: (Pre-treatment THI score - 3-month post-treatment THI score) / Pre-treatment THI score × 100%.

### 2.3 Stimulation

All interventions were performed using a Magstim Rapid2 stimulator with a 70-mm figure-of-eight coil, and Brainsight neuronavigation was used for precise targeting. Heschl’s gyrus (primary auditory cortex) and posterior superior temporal gyrus were selected as target regions, given that these regions constitute key substrates for tinnitus-related phantom sound perception and pathological cortical hyperexcitability (17). The exact stimulation site was localized to the peak activation voxel within these regions, based on individual activation maps from our event-related auditory task-fMRI paradigm (full acquisition and analysis details are provided in Sections 2.4 and 2.5). For unilateral tinnitus, the contralesional hemisphere was stimulated; for bilateral tinnitus, the left hemisphere was stimulated uniformly (14).

*rTMS group*: 1 Hz, 100–110% resting motor threshold (RMT), 1,200 pulses/session (20 min), once daily, 10 sessions over 2 weeks (total 12,000 pulses) (18).
*cTBS group*: 3-pulse bursts at 50 Hz repeated at 5 Hz, 80% RMT, two sessions daily (600 pulses/session, 40 s/session, 15-min interval), 10 consecutive days (total 12,000 pulses) (19).

RMT was measured according to international standards: the minimal intensity eliciting ≥50 μV motor-evoked potentials in at least 3 out of 5 consecutive stimulations over the contralateral first dorsal interosseous muscle.

### 2.4 MRI data acquisition

All participants underwent MRI scanning before and after the treatment course using a GE SIGNA Architect 3.0 T superconducting MRI scanner equipped with a 48-channel head coil. The scanning protocol included a high-resolution 3D T1-weighted sequence and a resting-state functional MRI (rs-fMRI) sequence and a task-based functional MRI (task-fMRI) sequence. The 3D T1-weighted images were acquired using a Magnetization Prepared Rapid Gradient Echo (MPRAGE) sequence with the following parameters: repetition time (TR) = 2.678 s, echo time (TE) = 2.98 ms, inversion time (TI) = 1.1 s, flip angle = 7°, slice thickness = 1.0 mm, slice gap = 1.0 mm, acquisition matrix = 256 × 256, and a parallel imaging acceleration factor of 2; these structural images were used for subsequent neuronavigation and spatial normalization. The rs-fMRI data were acquired using a Gradient-Recalled Echo Echo-Planar Imaging (GRE-EPI) sequence with parameters: TR = 1,000 ms, TE = 30 ms, flip angle = 80°, slice thickness = 3.0 mm, slice gap = 3.0 mm, and a multiband acceleration factor of 4; The full rs-fMRI scan lasted 8 min, with a total of 480 functional volumes collected. During rs-fMRI acquisition, participants were instructed to keep their eyes closed, stay awake, maintain a still supine position, and refrain from deliberate focused thinking.

For task-fMRI, an event-related auditory paradigm was adopted. The scan lasted 8 min 13 s, generating a total of 247 volumes. An interleaved acquisition scheme (1 s volume acquisition followed by a 1 s silent interval) was implemented to minimize interference from gradient acoustic noise and improve the discriminability of auditory stimuli inside the scanner environment. The paradigm contained 60 auditory stimuli, including 30 pure tone (PT) stimuli and 30 white noise (WN) stimuli. Each sound stimulus lasted 1 s. Pseudo-randomized inter-stimulus intervals of 4 s, 6 s, and 8 s were inserted between trials. Participants were required to press button “1” when hearing sounds resembling their tinnitus percept, and press button “2” for all other stimuli. Reaction time and response accuracy for each trial were recorded using E-prime 2.0 (Psychology Software Tools, Inc.) (20).

### 2.5 Data analysis

Clinical data were analyzed using SPSS (Version 26.0; IBM, Armonk, NY, USA). Rs-fMRI data and task-fMRI data were preprocessed and analyzed using MATLAB (MathWorks, Natick, MA, USA) and DPABI toolbox (version 8.0) (21).

Resting-state fMRI data were preprocessed using the DPARSF toolbox. The first 10 volumes were discarded to ensure steady-state magnetization. The preprocessing pipeline included: (1) slice-timing correction, (2) head-motion correction (Friston 24-parameter model to regress out head-motion effects), (3) nuisance regression (regressing out signals from white matter and cerebrospinal fluid), (4) spatial normalization to the MNI template (resampled to 3 mm cubic voxels), (5) spatial smoothing (6 mm FWHM Gaussian kernel), and (6) temporal band-pass filtering (0.01–0.08 Hz) to remove high-frequency noise and low-frequency drift.

To disentangle focal cortical changes and large-scale network reorganization induced by neuromodulation, we adopted three rs-fMRI metrics. Amplitude of Low-Frequency Fluctuations (ALFF) quantifies the magnitude of spontaneous regional neural activity (22), while Regional Homogeneity (ReHo) reflects the temporal synchrony of neurons within discrete brain regions. Seed-based functional connectivity (FC) further evaluates coordinated signal interactions between spatially separated brain areas to characterize whole-brain network communication (23).

ALFF and ReHo values were calculated for each voxel. Paired samples *t*-tests were used to compare pre- and post-treatment differences within groups, with GRF correction (voxel *p* < 0.005, cluster *p* < 0.05, cluster size ≥20 voxels). A 2 (group: cTBS vs. rTMS) × 2 (time: pre-treatment vs. post-treatment) full-factorial ANCOVA was performed within SPM12. Age, sex, tinnitus duration and head motion parameters were entered as nuisance covariates to eliminate confounding effects. Statistical threshold was set at voxel-level *p* < 0.001 uncorrected with a minimum cluster size of 18 voxels.

Task-fMRI data were processed to define individual stimulation target coordinates. Preprocessing of task-fMRI was conducted via DPABI, consisting of: (1) DICOM to NIfTI format conversion; (2) removal of the initial six volumes; (3) slice-timing correction, aligning all slices to the middle slice; (4) realignment to the mean functional volume with resampling; (5) spatial normalization using the DARTEL algorithm to warp individual images into standard MNI space, resampled to 3 × 3 × 3 mm voxels; (6) spatial smoothing with a 6 mm FWHM Gaussian kernel.

Single-subject activation analysis was performed in SPM12 within the general linear model (GLM). Regressors for pure tone stimuli, white noise stimuli, and six head-motion parameters (three translational and three rotational parameters) were incorporated into the design matrix. The contrast of tinnitus-matched sound versus alternative sound was computed to generate individual T-maps reflecting brain activation elicited by tinnitus-like auditory stimuli.

Mask-restricted activation analysis combined SPM12 and DPABI following these steps: (1) manual adjustment of the origin of T1-weighted images to the anterior commissure, ensuring the anterior and posterior commissures were horizontally aligned on the sagittal plane; (2) segmentation of T1 images to generate forward and inverse transformation matrices between native individual space and MNI standard space; (3) transformation of standard-space masks of the left anterior cingulate cortex and left DLPFC, together with individual activation maps, into native space; (4) resampling of masks and activation maps to identical spatial resolution; (5) multiplication of masks and individual activation maps to extract activation information confined within predefined anatomical regions.

Individual therapeutic target coordinates were determined in DPABI by identifying the peak activation voxel within each mask. If multiple activation peaks existed inside a mask, the peak closest to the scalp surface was selected as the final stimulation target.

Based on our task-fMRI results, we identified the left superior temporal gyrus (STG, cTBS: MNI: −65, −11, 4; rTMS: MNI: −60, −37, 17) as the primary hub of tinnitus-related neural activity. This cluster was utilized as the ROI (Region of Interest) for subsequent whole-brain functional connectivity (FC) analysis. For each subject, the mean time series was extracted from the seed ROI, and Pearson’s correlation coefficients were calculated between the seed time series and the time series of every other voxel in the brain. These correlation coefficients were converted into *z*-scores using Fisher’s r-to-z transformation to improve normality. Finally, paired *t*-tests were employed to compare the *z*-value maps between pre- and post-treatment states within each group. The results were corrected for multiple comparisons using the Gaussian Random Field (GRF) method (voxel *p* < 0.005, cluster *p* < 0.05, cluster size ≥20 voxels).

Mixed-design ANOVA was used for clinical efficacy comparison. Spearman rank correlation analysis was used to explore the association between tinnitus duration and THI improvement rate.

### 2.6 Baseline comparability

Independent samples t-tests and chi-square tests were used to compare baseline characteristics between groups. *p* > 0.05 indicated no significant difference, though interpretation should be cautious given the limited sample size.

## 3 Results

### 3.1 Comparability analysis of baseline data

There were no significant differences between the two groups in terms of age, tinnitus duration, sex composition, or distribution of tinnitus laterality. There was no significant difference in terms of hearing levels between the two groups. Furthermore, there were no significant differences in pre-treatment tinnitus severity indicators (THI scores, VAS scores) between the two groups, indicating good comparability at baseline in Table 1.

**Table 1:** Comparison of demographic characteristics and baseline clinical data between the two groups.

Variables	rTMS (*n* = 13)	cTBS (*n* = 16)	*p*-value
Age (years)	43.23 ± 11.22	39.12 ± 12.12	0.355
Sex (male/female)	9/4	10/6	1.000
Duration (months)	60.09 ± 17.44	66.00 ± 16.24	0.660
Tinnitus sides (L/R/B)	4/3/6	2/3/11	0.397
Left ear PTA (dB HL)	26.77 ± 11.46	26.65 ± 8.16	0.975
Right ear PTA (dB HL)	26.23 ± 13.51	24.96 ± 14.16	0.807
THI	45.54 ± 22.89	42.38 ± 18.65	0.683
VAS	5.80 ± 1.60	6.10 ± 1.45	0.602

Continuous variables (age, tinnitus duration, pure-tone average hearing threshold, THI, VAS scores) were compared using independent-samples t-tests. Categorical variables (sex, tinnitus side distribution) were compared using chi-square tests. Data are presented as mean ± standard deviation (x̄ ± s) or number (*n*). PTA, Pure-tone average; THI, Tinnitus Handicap Inventory; VAS, Visual Analogue Scale; *p*-value > 0.05 indicates no statistically significant difference between groups.

### 3.2 Clinical efficacy

The rTMS group showed a significant reduction in THI scores at the 3-month follow-up vs. baseline (*p* = 0.007). The THI scores in the cTBS group exhibited mild numerical reduction without statistically significant intertemporal change (*p* = 0.124). Mixed-design ANOVA revealed a significant time × group interaction (*F*(3,81) = 3.05, *p* = 0.033).

The cTBS group exhibited a statistically significant reduction in VAS scores (*p* = 0.004), but the effect was not maintained at 1 month follow-up and 3 month follow-up (*p* = 0.082, *p* = 0.117). The rTMS group showed no significant VAS changes (post-treatment: *p* = 0.214; 1 month: *p* = 0.307; 3 month: *p* = 0.332). A significant time × group interaction was observed (*F*(3,81) = 3.98, *p* = 0.011) (Figure 1 and Table 2).

**Figure 1:**
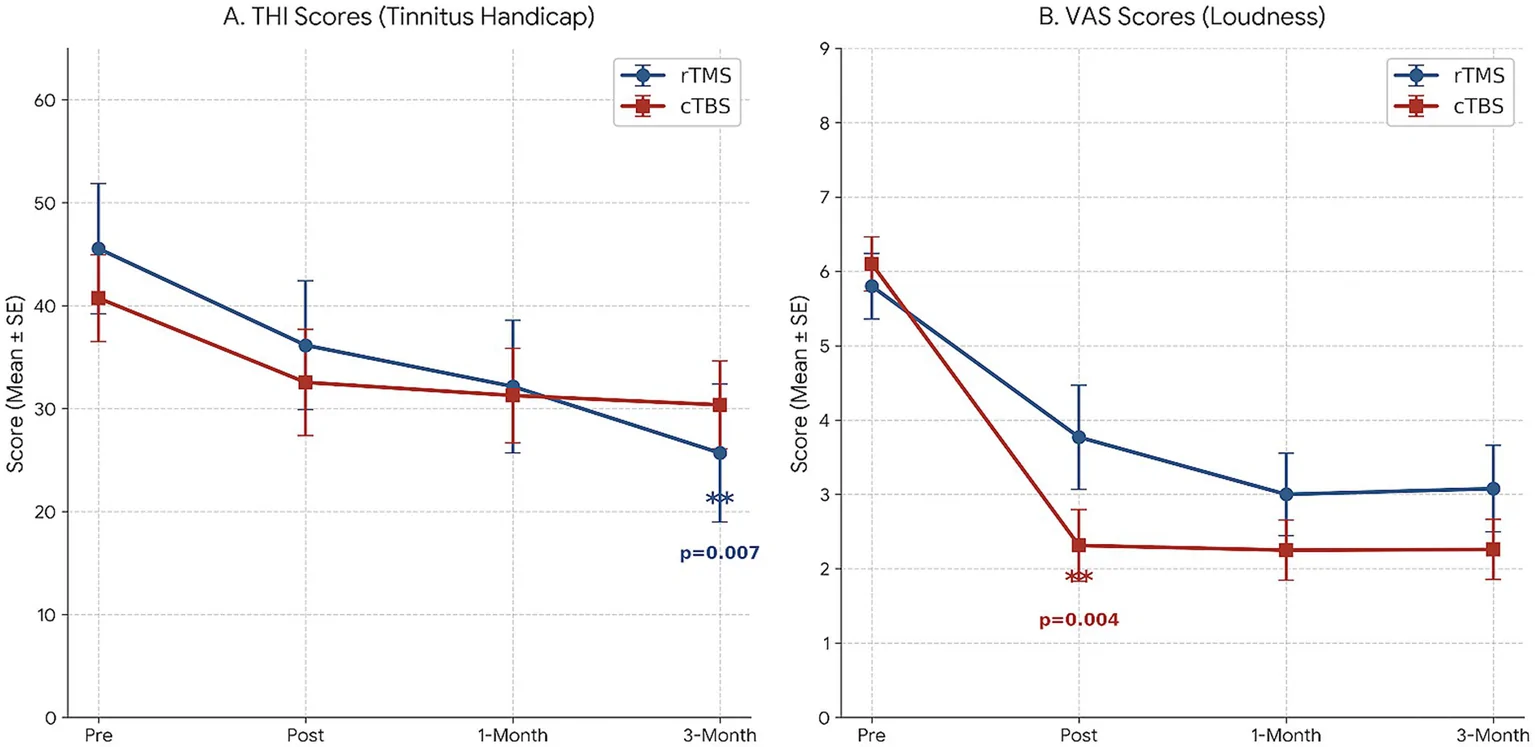
Comparison of THI and VAS scale follow-up results with baseline values. The left panel shows the change in Tinnitus Handicap Inventory (THI) scores over time; the right panel shows the change in Visual Analogue Scale (VAS) scores. Data are presented as mean ± standard error (x̄ ± s). *p* < 0.01 indicates a statistically significant difference compared to baseline (based on repeated measures ANOVA and Bonferroni *post-hoc* tests).

**Table 2:** Change in the average THI and VAS score between before and after treatment in both groups of tinnitus patients.

Variables	Group	Baseline	Post-treatment	1-month	3-month	*p*-value (Time × Group)
THI	rTMS	45.54 ± 22.89	36.25 ± 21.32	32.35 ± 19.58	25.77 ± 15.22*	0.033
	cTBS	42.38 ± 18.65	32.69 ± 15.40	31.31 ± 13.52	30.38 ± 14.21	
VAS	rTMS	5.80 ± 1.60	3.77 ± 1.85*	3.00 ± 1.78	3.08 ± 1.82	0.011
	cTBS	6.10 ± 1.45	2.31 ± 0.95*	2.25 ± 0.85	2.25 ± 0.85	

* indicates statistically significant differences within the group compared to baseline (THI in the rTMS group at 3 months, *p* = 0.007; VAS at post-treatment in the cTBS group, *p* = 0.004).

### 3.3 Local brain-activity changes (ALFF and ReHo)

Significantly decreased ALFF in the left superior and inferior temporal gyri; significantly increased ReHo in the bilateral superior temporal gyri and left middle temporal gyrus.

Significantly increased ALFF in the left anterior cingulate cortex, right inferior frontal gyrus, and left parahippocampal gyrus; significantly decreased ReHo in the precuneus and left superior frontal gyrus. Significant clusters were defined as those surviving the GRF correction (voxel-level *p* < 0.005, cluster-level *p* < 0.05). The peak t-values and cluster sizes (voxels) for these surviving regions are detailed in Table 3 and Figures 2, 3.

**Table 3:** Distribution of brain regions with significant brain activity changes induced by cTBS and rTMS.

Group	Indicator	Brain region	MNI (x, y, z)	*T*	Change
cTBS	ALFF	Left inferior temporal gyrus (L-ITG)	−51, −15, −33	−3.923	Decrease ↓
cTBS	ALFF	Left superior temporal gyrus (L-STG)	−57, −3, 0	−5.342	Decrease ↓
cTBS	ReHo	Left superior temporal gyrus (L-STG)	−54, −21, 21	6.064	Increase ↑
cTBS	ReHo	Left middle temporal gyrus (L-MTG)	−57, −24, −9	5.196	Increase ↑
cTBS	ReHo	Right superior temporal gyrus (R-STG)	54, −15, 3	4.234	Increase ↑
rTMS	ALFF	Right inferior frontal gyrus (R-IFG)	9, 12, −21	5.010	Increase ↑
rTMS	ALFF	Left anterior cingulate cortex (L-ACC)	−18, 18, 21	5.509	Increase ↑
rTMS	ALFF	Left parahippocampal gyrus (L-PHG)	−21, −33, −9	5.368	Increase ↑
rTMS	ReHo	Left Precuneus	3, −51, 30	−4.017	Decrease ↓
rTMS	ReHo	Left superior frontal gyrus (L-SFG)	−9, 60, 9	−4.379	Decrease ↓

L, Left; R, Right. MNI, Montreal Neurological Institute standard space. T, Peak intensity. Paired sample *t*-tests were performed on pre- and post-treatment ALFF and ReHo values for all subjects. Results were corrected using GRF, with thresholds set at Voxel *p* value < 0.005, cluster *p* value < 0.05 and a minimum cluster size of 20 voxels.

**Figure 2:**
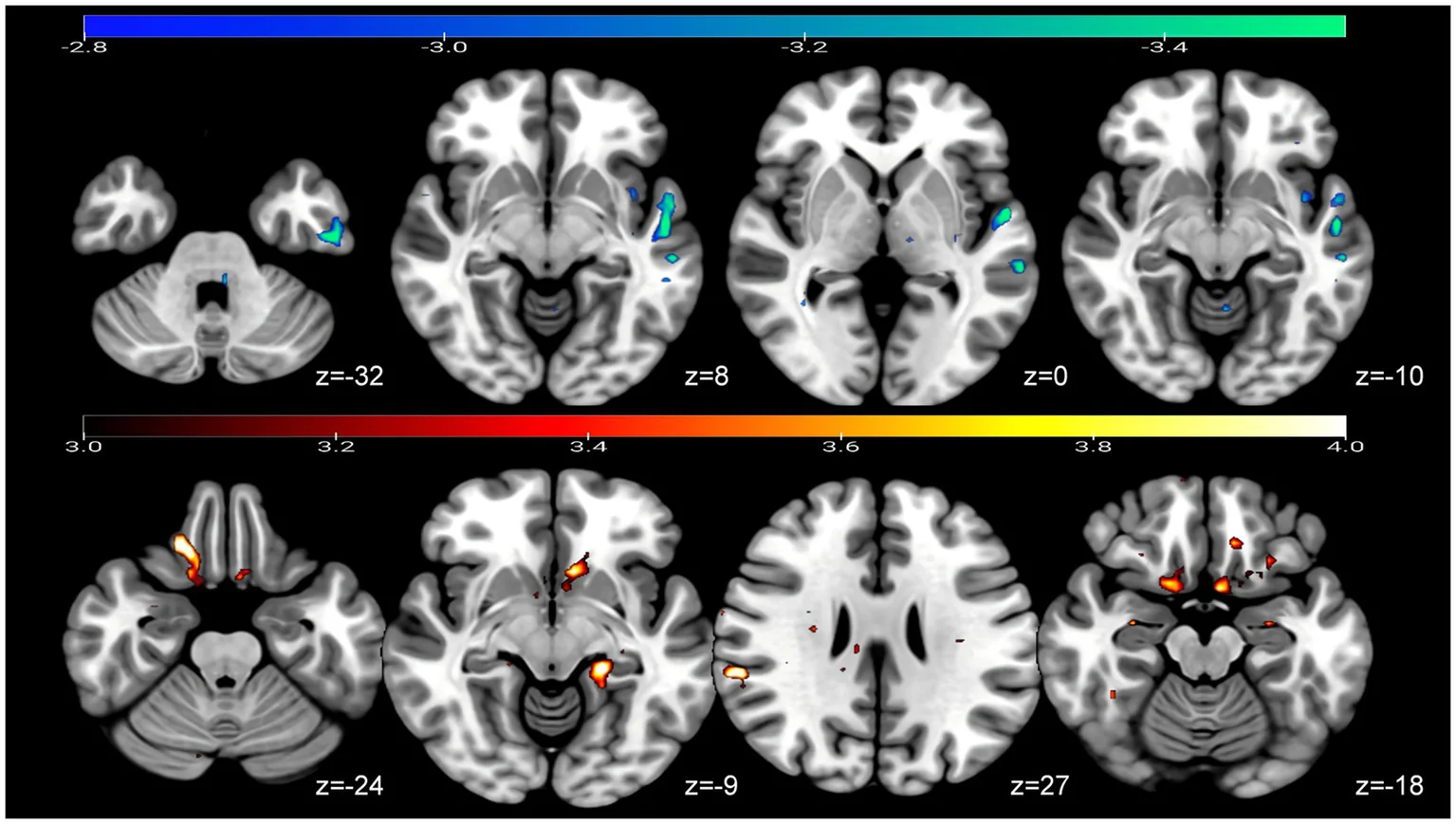
Distribution of brain regions with ALFF changes induced by cTBS and rTMS treatment. The upper panel displays the ALFF changes before and after cTBS treatment, while the lower panel shows the ALFF changes before and after rTMS treatment. In the color bar, blue indicates a decrease and red indicates an increase in ALFF values compared to pre-treatment. The background is the MNI152 standard brain template.

**Figure 3:**
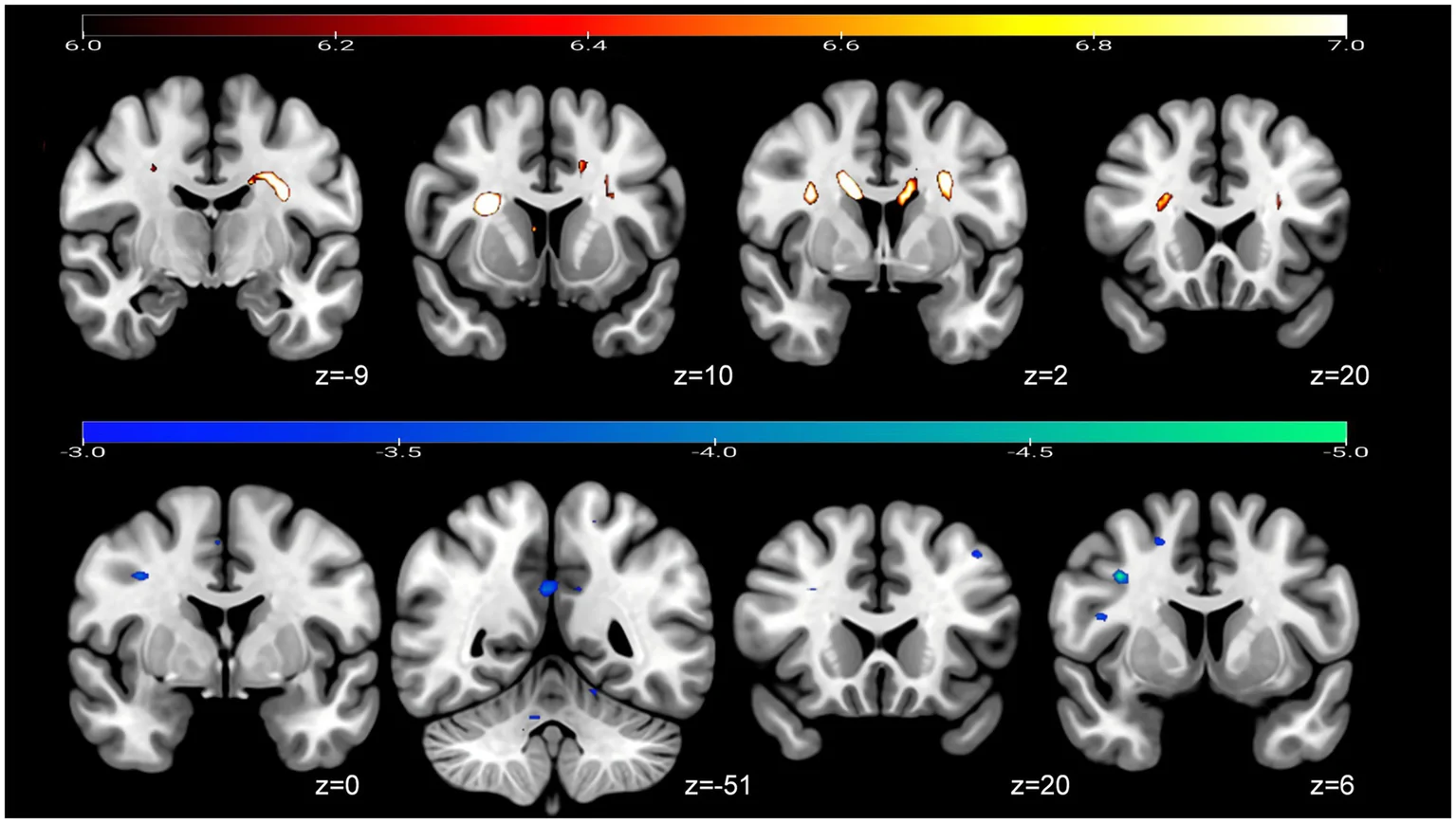
Distribution of brain regions with ReHo changes induced by cTBS and rTMS treatment. The upper panel displays the ReHo changes before and after cTBS treatment, while the lower panel shows the ReHo changes before and after rTMS treatment. In the color bar, blue indicates a decrease and red indicates an increase in ReHo values compared to pre-treatment. The background is the MNI152 standard brain template.

Two-way ANCOVA identified a significant group × time interaction cluster in the left prefrontal cortex (MNI coordinates: −30, 24, −18, peak *T* = 4.69) across both ALFF and ReHo analyses (Figure 4).

**Figure 4:**
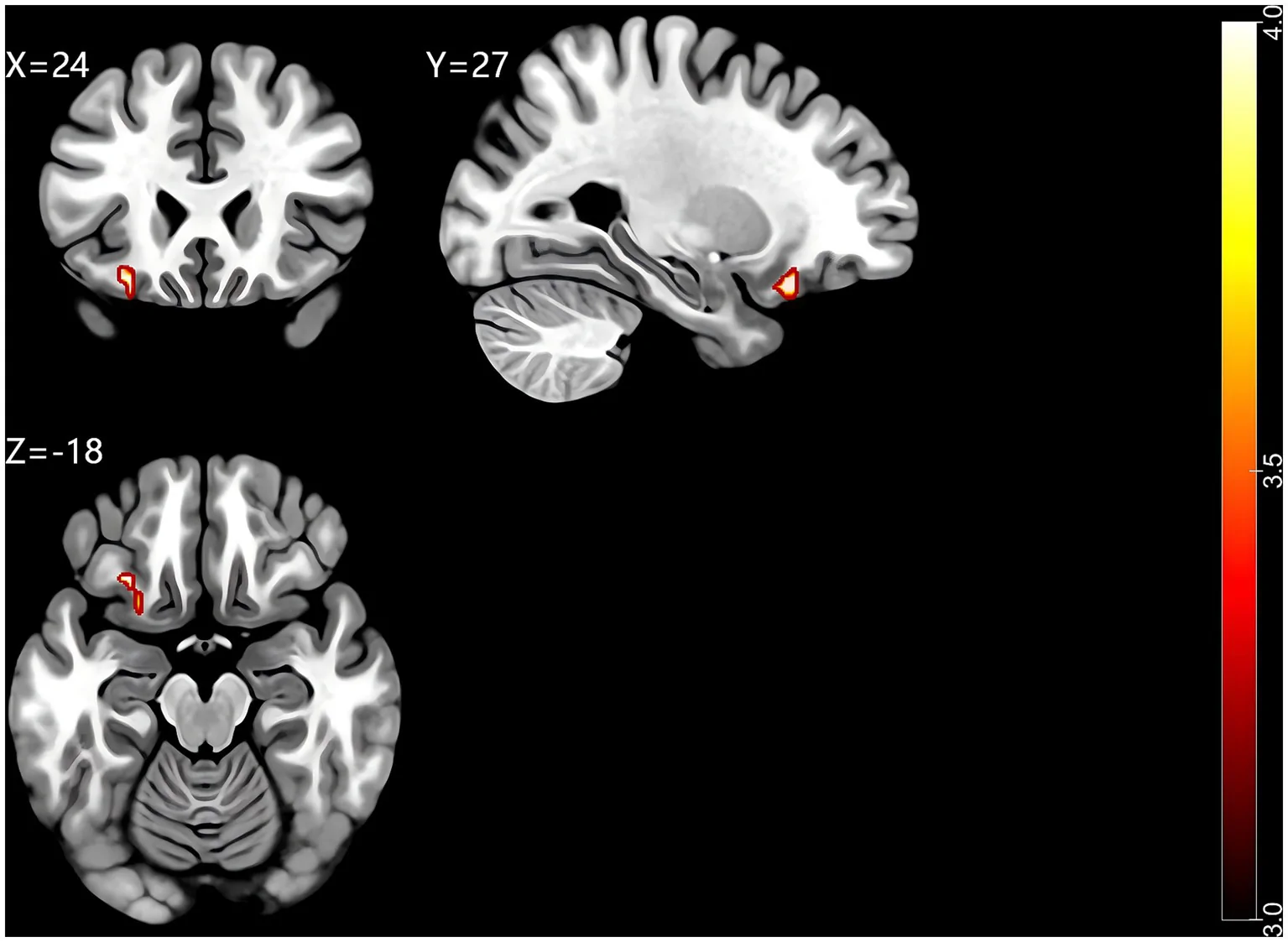
Significant group-by-time interaction clusters of ALFF and ReHo between cTBS and low-frequency rTMS groups. The panels show the sagittal, coronal and axial views of the consistent significant group-by-time interaction cluster in the left prefrontal cortex detected from both ALFF and ReHo analyses. The color bar represents *T*-statistic values of the interaction contrast. The background is the MNI152 standard brain template.

### 3.4 Functional connectivity changes

To further characterize network-level effects of the two TMS protocols, seed-based functional connectivity (FC) analysis was performed using the left superior temporal gyrus as the seed. The cTBS group showed significant increases in FC within the left middle occipital gyrus, left superior frontal gyrus, and right angular gyrus (Table 4). Conversely, the rTMS group demonstrated extensive network remodeling, including increased connectivity in the left postcentral gyrus, and decreased connectivity in the left precuneus, right superior parietal lobule, and right inferior temporal gyrus (Table 5; Figure 5).

**Table 4:** Distribution of brain regions showing significant changes in FC values before and after cTBS treatment.

Brain region	BA	MNI			Voxels	*T*	Changes
		X	Y	Z			
Left Middle Occipital Gyrus (L-MOG)	BA19_L	−30	−75	36	45	5.18	Increase ↑
Left Superior Frontal Gyrus (L-SFG)	BA8_L	−21	−45	21	25	4.35	Increase ↑
Right Angular Gyrus (RAG)	BA39_R	39	−63	33	20	3.79	Increase ↑

The ROI of the left superior temporal gyrus is (−65, −11, 4). Paired *t*-tests were performed on whole-brain functional connectivity (FC) values across all subjects with tinnitus before and after cTBS treatment, with results corrected for GRF. Results were corrected using GRF, with thresholds set at Voxel *p* value < 0.005, cluster *p* value < 0.05 and a minimum cluster size of 20 voxels.

**Table 5:** Distribution of brain regions showing significant changes in FC values before and after rTMS treatment.

Brain region	BA	MNI			Voxels	*T*	Changes
		X	Y	Z			
Postcentral Gyrus Left (PoCG.L)	BA4/6_L	−66	−9	24	58	5.97	Increase ↑
Left Precuneus	BA7/4_L	3	−39	72	45	−4.68	Decrease ↓
Right Superior Parietal Lobule (R-SPL)	BA7_R	21	−75	57	31	−3.98	Decrease ↓
Right Inferior Temporal Gyrus (R-ITG)	BA37_R	48	−54	12	27	−4.41	Decrease ↓

The ROI of the left superior temporal gyrus is (−60, −37, 17). Paired *t*-tests were performed on whole-brain functional connectivity (FC) values across all subjects with tinnitus before and after rTMS treatment, with results corrected for GRF. Results were corrected using GRF, with thresholds set at Voxel *p* value < 0.005, cluster *p* value < 0.05 and a minimum cluster size of 20 voxels.

**Figure 5:**
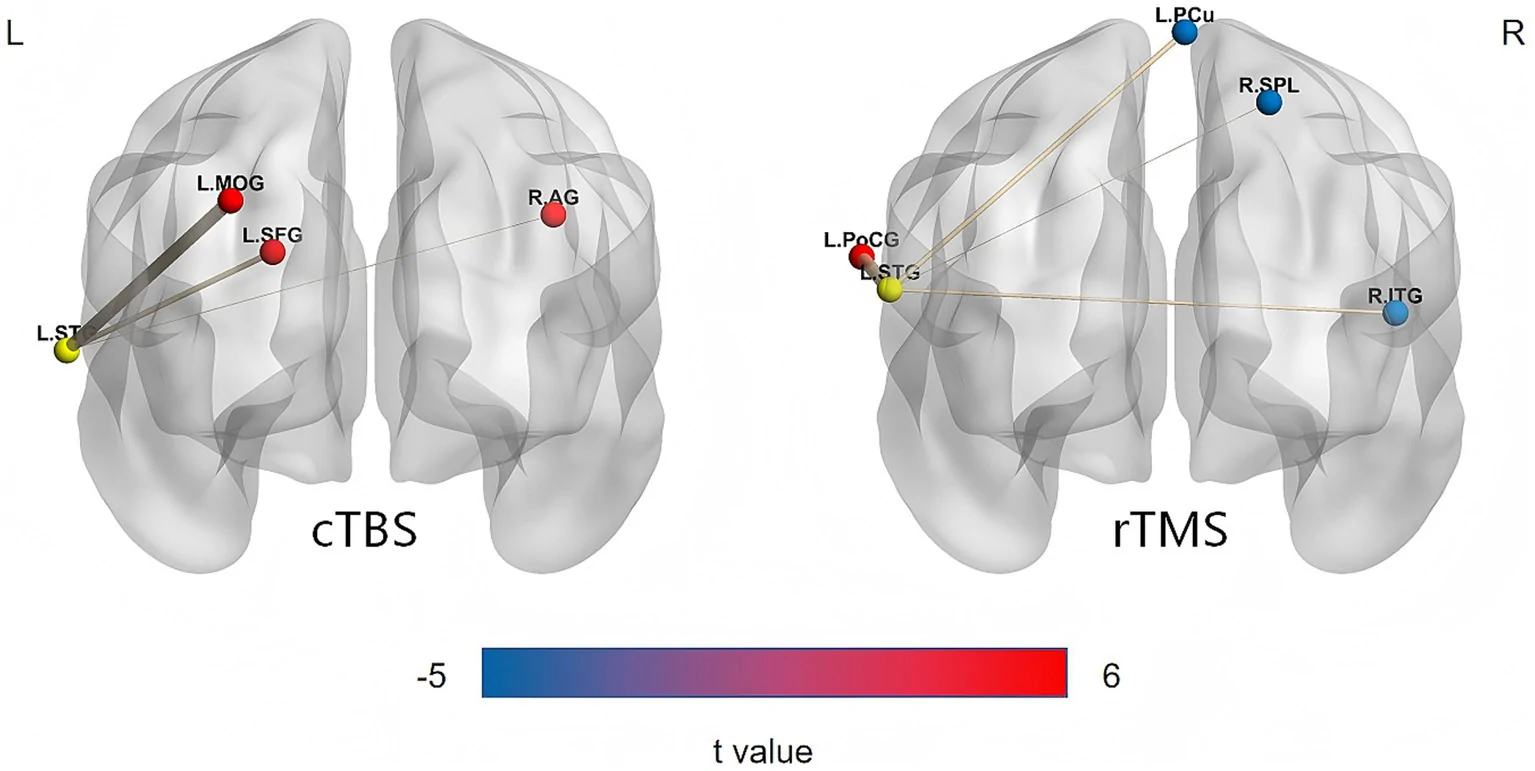
Modulation of whole-brain functional connectivity (FC) patterns following cTBS and rTMS interventions. The color bar represents the range of *t*-values, with red and blue indicating increases and decreases in functional connectivity, respectively. The yellow spheres represent seed region. The thickness of the yellow lines represents the magnitude of the *t*-value for the connection between the seed region and each of these brain regions. L, left; R, right; STG, Superior Temporal Gyrus; MOG, Middle Occipital Gyrus; SFG, Superior Frontal Gyrus; AG, Angular Gyrus; PoCG, Postcentral Gyrus; PCu, Precuneus; SPL, Superior Parietal Lobule; ITG, Inferior Temporal Gyrus.

### 3.5 Correlation analysis between disease duration and treatment efficacy

Exploratory Spearman correlation showed a significant negative correlation between tinnitus duration and 3-month THI improvement rate (*r* = −0.45, *p* = 0.013). Multiple linear regression showed no significant independent predictive effect of any variable (*p* = 0.556), likely due to limited statistical power (Figure 6).

**Figure 6:**
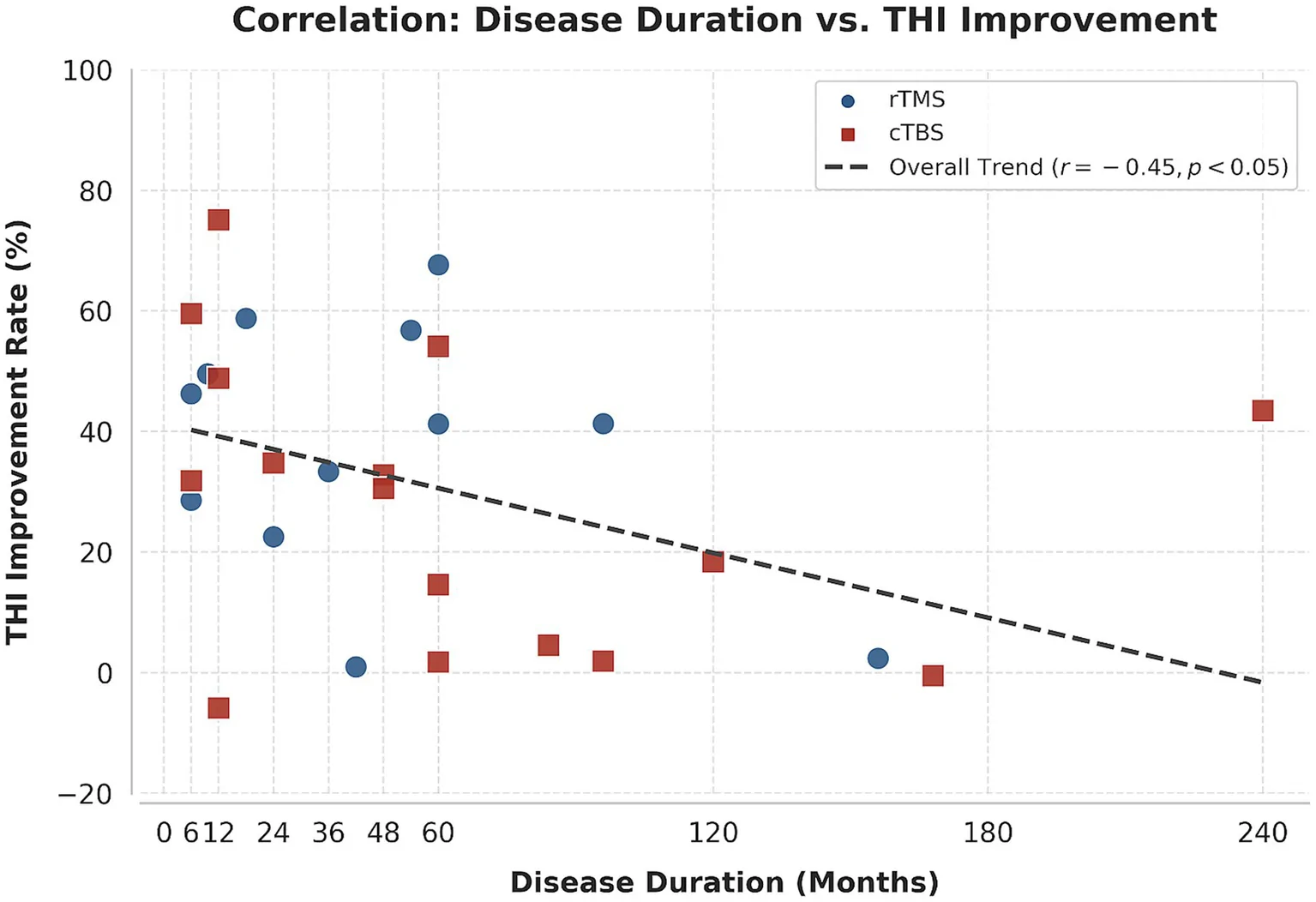
Scatter plot of correlation analysis between disease duration and efficacy. The horizontal axis represents tinnitus duration (months), and the vertical axis represents the THI improvement rate (%) post-treatment. Blue circles represent patients in the rTMS group, and red squares represent patients in the cTBS group. The black dashed line is the overall fitting trend line.

## 4 Discussion

This preliminary exploratory study directly compared cTBS and low-frequency rTMS for chronic tinnitus, revealing dissociable efficacy patterns and neural modulation trends. Our exploratory results suggest that cTBS may facilitate a rapid reduction in tinnitus loudness predominantly through focal auditory-cortical modulation, while rTMS gradually alleviates tinnitus-related distress through more extensive network-level limbic-cognitive remodeling. Tinnitus duration was preliminarily associated with treatment response, supporting the potential value of early intervention.

### 4.1 Symptom-specific efficacy: loudness vs. distress

The divergent clinical response patterns observed in our study support the “multi-dimensional network model” of tinnitus, which posits that loudness and distress are governed by distinct, albeit interacting, neural circuits (24).

cTBS produced a statistically significant reduction in VAS loudness scores immediately post-treatment (*p* = 0.004). We speculate that this rapid “front-loaded” efficacy may originate from its high-frequency burst-stimulation pattern, which could effectively dampen pathological high-frequency gamma-band oscillations within core auditory centers, consistent with pathological models of tinnitus-related auditory-cortex hyper-synchrony (25). The present study suggests that direct and robust inhibition of hypersynchronized activity in the auditory cortex is required to reduce the perceptual loudness of tinnitus (26). Consistently, significant alterations in auditory cortical ALFF and ReHo values in the cTBS group indicate that burst theta stimulation interferes with pathological neural oscillations more potently than conventional low-frequency rTMS, thereby achieving immediate suppression of tinnitus loudness. Nevertheless, although THI scores showed a declining trend following cTBS, the change failed to reach statistical significance, and the sustained up to 3 months influence of cTBS on tinnitus distress awaits validation in larger cohorts.

By contrast, low-frequency rTMS yielded a statistically significant reduction in THI at the 3-month follow-up (*p* = 0.007), without prominent acute effects on perceived loudness. Previous meta-analyses similarly demonstrated that rTMS preferentially improves THI rather than VAS outcomes in tinnitus patients (27), implying rTMS mainly modulates patients’ emotional responses and tolerance to tinnitus, instead of directly eliminating auditory phantom percepts. The sustained improvement in tinnitus distress aligns with our imaging findings of altered activity within emotion-related regions, including the parahippocampal gyrus (linked to emotional memory) and anterior cingulate cortex (associated with affective distress). This clinical pattern preliminarily supports the categorization of cTBS as a “focal suppressor” targeting perceptual loudness and rTMS as a “network remodeler” that alleviates tinnitus-related emotional burden.

### 4.2 Neural modulation patterns: localized focusing vs. network-level dispersion

Our multimodal resting-state fMRI results provide mechanistic explanations for the differentiated clinical efficacy of the two neuromodulation protocols. We found that modulatory effects induced by cTBS were highly localized to the superior temporal gyrus (STG) and middle temporal gyrus (MTG). Earlier PET investigations also confirmed that cTBS markedly reduces local cerebral blood flow beneath the stimulation target (28), and our ALFF and ReHo data further extend this observation. Regulation of local signal synchronization (ReHo) and spontaneous activity intensity (ALFF) within primary auditory cortices may directly disrupt tinnitus-associated pathological neural oscillations, constituting a plausible neural substrate for the rapid reduction of subjective loudness after cTBS intervention. Of note, cTBS also elicited limited remote functional-connectivity changes involving the middle occipital gyrus and several other discrete cortical nodes. Such distal connectivity shifts are expected following focal cortical TMS, as stimulation-evoked plasticity can propagate along intrinsic anatomical pathways. Nevertheless, these remote alterations were sparse and did not engage core limbic-cognitive or default-mode-network hubs, distinguishing this pattern from the broad, system-spanning network remodeling observed in the rTMS group.

In contrast, although low-frequency rTMS targets the same auditory cortex, its significant brain alterations emerged widely across distributed networks beyond the stimulation site. Seed-based functional connectivity (FC) analysis revealed extensive network reorganization in the rTMS group: strengthened connectivity was detected in the left postcentral gyrus, while reduced connectivity was observed in the left precuneus, right superior parietal lobule, and right inferior temporal gyrus. Significant remodeling spanned the limbic system (e.g., parahippocampal gyrus), default mode network (DMN; e.g., precuneus), prefrontal cognitive control regions, and thalamus. These findings support the “network-level dispersion” hypothesis proposed by De Ridder et al. (29). Through prolonged repetitive single-pulse stimulation, rTMS propagates inhibitory influences across interconnected brain networks to engage limbic and DMN circuits. The detected functional changes in the precuneus and parahippocampal gyrus appear to align with the “noise-canceling deficit model” (30): rTMS partially restores the impaired auditory-limbic-prefrontal gating circuit, which not only suppresses aberrant auditory signals but also blocks their conversion into persistent negative emotional awareness.

### 4.3 Divergent inhibitory profiles validated by group-by-time interaction analysis

Beyond separate within-group pre–post comparisons, the group-by-time interaction ANCOVA offered direct statistical evidence of divergent inhibitory strength between cTBS and rTMS at prefrontal cognitive-emotional hubs. As a core node within the auditory-limbic-prefrontal gating circuit described by the noise-canceling deficit model, the left prefrontal cortex mediates top-down control over tinnitus-related negative emotion and abnormal auditory processing (31). The stronger suppressive modulation exerted by cTBS within this prefrontal region matches its intrinsic focal inhibitory property. After suppressing auditory cortex hyperactivity, this inhibitory effect propagates to upstream prefrontal regulatory areas. On the other hand, low-frequency rTMS relies on serial slow single pulses to modulate large-scale brain networks, leading to mild focal inhibition in the prefrontal cortex and distributed functional remodeling across limbic and DMN circuits.

Notably, consistent group-by-time interaction patterns were identified across both ALFF and ReHo metrics, which quantify local spontaneous activity amplitude and inter-voxel signal synchronization, respectively. Concordant outcomes from two independent resting-state measurements rule out the possibility that group differences originated from random physiological noise or bias associated with a single imaging index, reinforcing our core framework: cTBS exerts localized focal modulation, whereas low-frequency rTMS drives dispersed network remodeling. The absence of significant interaction clusters within the primary auditory cortex further indicates that both inhibitory interventions generate comparable direct suppressive effects on stimulated temporal targets; their functional divergence primarily manifests in downstream higher-order brain regions responsible for emotion and cognition.

### 4.4 Tinnitus duration as a potential prognostic factor

The exploratory negative correlation between tinnitus duration and THI improvement suggests that patients with shorter tinnitus duration may benefit more from neuromodulation, possibly due to more flexible neuroplasticity. However, the non-significant multiple regression model indicates that the independent predictive role of duration remains unconfirmed and requires validation in large-sample studies.

## 5 Limitations

Several critical limitations must be addressed to appropriately contextualize our findings. First, and most importantly, this study lacked a sham-controlled arm. Consequently, the observed clinical and fMRI-based improvements cannot be fully distinguished from placebo effects, non-specific contextual stimulation effects, or the natural fluctuations of tinnitus symptoms over time. All mechanistic claims regarding the neural signatures of cTBS and rTMS are exploratory and correlational; therefore, causal attributions of symptom relief to the active neuromodulation remain unsubstantiated and require validation in future sham-controlled, double-blinded trials.

Second, the sample size was modest (*n* = 29). Although our post-hoc power analysis (Supplementary Table 1) quantified the statistical limitations, the lack of sufficient power for multiple linear regression likely constrained our ability to identify disease duration as a definitive prognostic predictor. Our results should be interpreted as hypothesis-generating, providing a preliminary framework rather than clinical guidance for routine practice.

Third, our study design involved a mismatch in stimulation scheduling: the rTMS group received once-daily sessions, while the cTBS group received twice-daily sessions. While total pulse counts were equalized to maintain magnetic dose consistency, the differential timing of stimulation delivery may independently modulate synaptic plasticity and could function as a confounding variable. Future protocols should standardize the stimulation schedule to isolate the effects of the stimulation patterns themselves.

Finally, while our longitudinal data demonstrate that therapeutic gains were sustained up to the 3-month follow-up, the long-term clinical durability beyond this timeframe remains unknown. Further research with extended follow-up periods (6–12 months) is essential to determine the sustainability of these interventions and to indicate whether cTBS and rTMS serve distinct, complementary roles in personalized tinnitus neuromodulation.

## 6 Conclusion

In this preliminary exploratory study, cTBS and low-frequency rTMS suggest distinct efficacy profiles and neural modulation patterns when applied to the auditory cortex in chronic tinnitus. Consistent group × time interaction effects across both ALFF, ReHo and FC analyses further support the hypothesis of divergent suppressive neuromodulation strength between the two inhibitory protocols within the left prefrontal cortex, a core hub of the auditory-limbic-prefrontal top-down gating circuit for tinnitus emotion regulation. cTBS may be associated with rapid, transient relief of tinnitus loudness via localized modulation of core auditory regions, while low-frequency rTMS may provide gradual improvement of tinnitus-related distress through network-level remodeling of the limbic-cognitive system. Tinnitus duration is preliminarily associated with treatment efficacy, highlighting the potential value of early intervention. While these improvements were sustained through our 3-month follow-up, future investigations with 6–12 month monitoring periods are required to establish true therapeutic durability. These findings provide exploratory mechanistic evidence for personalized neuromodulation in tinnitus, pending validation in large-sample, sham-controlled trials.

## Data Availability

The datasets generated and analyzed during this study are not publicly available, as they contain sensitive individual-level clinical information and resting-state functional MRI data from human participants. Data sharing is restricted by institutional ethical requirements and participant written informed consent, which prohibit unrestricted open distribution of identifiable neuroimaging data. Summary group-level statistical results are fully reported within the manuscript. De-identified raw data can be shared with qualified independent researchers upon reasonable formal request to the corresponding author. Data access will be subject to institutional review board approval and formal data confidentiality agreements. Requests to access the datasets should be directed to data access requests can be submitted to the corresponding author, YL, liyuan81629@163.com.
