# Supplementary material for: Differential mechanisms of continuous theta burst stimulation and low-frequency repetitive transcranial magnetic stimulation for chronic tinnitus: preliminary efficacy dissociation by local focalization vs. network dispersion
Source: Front Neurol. 2026 Sep 30;17:1921227. doi: 10.3389/fneur.2026.1921227 (PMC13661314; doi:10.3389/fneur.2026.1921227)
Supplement: Supplementary file 1 [file Table_1.DOCX]

Supplementary Table 1. Post-hoc Power Analysis for Key Findings

| Outcome Index | Statistical Test | Test Statistic | Degrees of Freedom (df₁, df₂) | p-value | Effect Size (Partial η² or r²) | Observed Power (α=0.05) |
| --- | --- | --- | --- | --- | --- | --- |
| THI Time× Group Interaction | Mixed-design ANCOVA | F = 3.05 | (3, 81) | 0.033 | 0.101 | 0.64 |
| VAS Time × Group Interaction | Mixed-design ANCOVA | F = 3.98 | (3, 81) | 0.011 | 0.129 | 0.78 |
| Tinnitus Duration ＆ THI Improvement | Spearman Correlation | r = -0.45 | N = 29 | 0.013 | 0.203 | 0.71 |

Note: Post-hoc statistical power was performed via G*Power 3.1.9.7 based on observed partial η² and correlation coefficients, with α = 0.05. Estimated power values < 0.8 indicate insufficient sample size to reliably detect small-to-moderate effects under the current cohort (N=29).
